# Potential of stable isotope analysis to deduce anaerobic biodegradation of ethyl *tert*-butyl ether (ETBE) and *tert*-butyl alcohol (TBA) in groundwater: a review

**DOI:** 10.1007/s11356-024-32109-3

**Published:** 2024-02-06

**Authors:** Marcelle J. van der Waals, Steven F. Thornton, Stephen A. Rolfe, Luc Rock, Jonathan W. N. Smith, Tom N.P. Bosma, Jan Gerritse

**Affiliations:** 1 Unit Subsurface and Groundwater Systems, Deltares, Daltonlaan 600, Utrecht, 3484 BK The Netherlands; 2 Present address: KWR Water Research Institute, Groningenhaven 7, 3433 PE Nieuwegein, The Netherlands; 3 Department of Civil and Structural Engineering, University of Sheffield, Mappin St, Sheffield, S1 3JD UK; 4 School of Biosciences, University of Sheffield, Western Bank, Sheffield, S10 2TN UK; 5 Shell Global Solutions International BV, Carel van Bylandtlaan 30, The Hague, 2596 HR The Netherlands; 6 Present address: Shell Global Solutions (Canada) Inc, 4000 – 500 Centre Street SE, Calgary, AB T2G 1A6 Canada; 7 Shell Global Solutions (UK) Ltd, Shell Centre, York Road, London, SE1 7NA UK

**Keywords:** Gasoline ether oxygenates, Tertiary alcohols, Isotopic fractionation, Compound-specific stable isotope analysis, Position-specific stable isotope analysis, Natural attenuation

## Abstract

**Abstract:**

Understanding anaerobic biodegradation of ether oxygenates beyond MTBE in groundwater is important, given that it is replaced by ETBE as a gasoline additive in several regions. The lack of studies demonstrating anaerobic biodegradation of ETBE, and its product TBA, reflects the relative resistance of ethers and alcohols with a tertiary carbon atom to enzymatic attack under anoxic conditions. Anaerobic ETBE- or TBA-degrading microorganisms have not been characterized. Only one field study suggested anaerobic ETBE biodegradation. Anaerobic (co)metabolism of ETBE or TBA was reported in anoxic microcosms, indicating their biodegradation potential in anoxic groundwater systems. Non-isotopic methods, such as the detection of contaminant loss, metabolites, or ETBE- and TBA-degrading bacteria are not sufficiently sensitive to track anaerobic biodegradation *in situ*. Compound- and position-specific stable isotope analysis provides a means to study MTBE biodegradation, but isotopic fractionation of ETBE has only been studied with a few aerobic bacteria (εC −0.7 to −1.7‰, εH −11 to −73‰) and at one anoxic field site (δ^2^H-ETBE +14‰). Similarly, stable carbon isotope enrichment (δ^13^C-TBA +6.5‰) indicated TBA biodegradation at an anoxic field site. CSIA and PSIA are promising methods to detect anaerobic ETBE and TBA biodegradation but need to be investigated further to assess their full potential at field scale.

**Graphical abstract:**

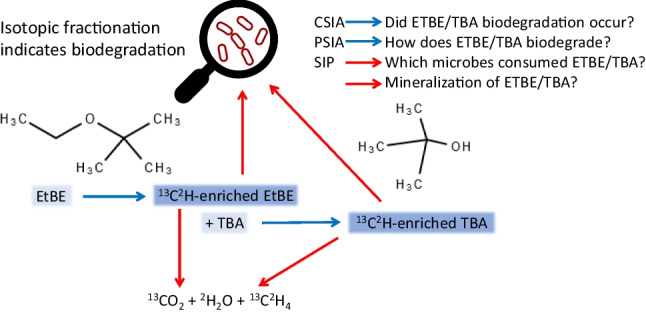

## Introduction

Ether oxygenates, such as methyl tert-butyl ether (MTBE), ethyl tert-butyl ether (ETBE), tert-amyl methyl ether (TAME), tert-amyl ethyl ether (TAEE), and diisopropyl ether (DIPE), are added to certain gasoline formulations to increase the octane number, thereby improving the efficiency of combustion and reducing emissions from vehicles (Fayolle et al. 2001; Concawe 2012; Thornton et al. 2020). ETBE is now the dominant ether oxygenate used in Europe and Japan, largely because it can be synthesized from biomass and contributes to the required renewable component of road fuel (van der Waals et al. 2019; Concawe 2020; Thornton et al. 2020).

ETBE can enter groundwater as a result of spillages during production, distribution, and storage. ETBE poses a hazard to groundwater resources because of its high aqueous solubility of 12 g/L at 20 °C (National Institutes of Health (n.d.)), its low thresholds for odour and taste in water (49 and 47 μg/L, respectively), and because little is known about its biodegradation potential in the subsurface (Babé et al. 2007; Le Digabel et al. 2013; van der Waals et al. 2019). The low taste and odour thresholds make ETBE impacted groundwater unusable for drinking purposes; however, the predicted no effect concentration (PNEC) and no observed effect concentration (NOEC) values are at least 1 order of magnitude greater than the taste and odour threshold values (reviewed in Thornton et al. 2020), meaning the primary focus is on aesthetic impacts. Hence, it is important to understand the fate of ETBE in the subsurface and how it could migrate to points of water use/discharge. Relatively few field-scale biodegradation studies have been published (Bombach et al. 2015; Nicholls et al. 2020; Thornton et al. 2020).

Tert-butyl alcohol (TBA) is a metabolite of MTBE and ETBE biodegradation, but also a potential fuel oxygenate (Piveteau et al. 2001). The biodegradation of MTBE under aerobic and anaerobic conditions has been studied intensively, and bacteria and biochemical pathways have been characterized (Deeb et al. 2003; Lopes Ferreira et al. 2006; Häggblom et al. 2007; Concawe 2012; Hyman 2013; Liu et al. 2016). Aerobic biodegradation is well-established for ETBE and TBA, but there is relatively little peer-reviewed science on the assessment, mechanisms, and extent of anaerobic ETBE or TBA biodegradation in the subsurface (Thornton et al. 2020). A limited number of studies suggest that TBA can be a key product of both the aerobic and anaerobic biodegradation of ETBE (Yeh and Novak 1994; Schmidt et al. 2004a, b; Concawe 2012; Thornton et al. 2020).

**Fig. 1:**
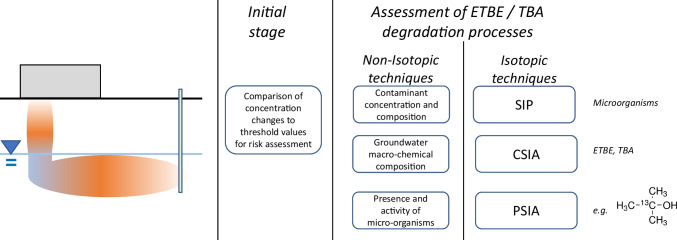
Summary of non-isotopic and isotopic techniques to detect ETBE and TBA biodegradation. The blue line in the figure indicates the groundwater level. The orange ovals indicate the contaminated area

Despite the increasing use of ETBE in gasoline, demonstrating its (and associated TBA) biodegradation potential *in situ* remains an important challenge to support natural attenuation strategies for site management and reducing environmental risk. This paper reviews the literature from laboratory and field studies on methods that can be used to demonstrate the biodegradation of ETBE and TBA in contaminated groundwater, with a focus on (stable) isotope approaches (Fig. 1). It also includes background information on the overall theme of assessing ETBE and TBA biodegradation in the environment.

## Natural attenuation of ETBE and TBA

Various processes may decrease ETBE and TBA concentrations in the subsurface. Dilution, sorption, volatilization, dispersion, and biological degradation (i.e., biodegradation, biological stabilization/destruction) can decrease concentrations in groundwater. These processes contribute to natural attenuation, which is defined by the USEPA as “a variety of physical, chemical, or biological processes that, under favorable conditions, act without human intervention to reduce the mass, toxicity, mobility, volume, or concentration of contaminants in soil or groundwater” (Bekins et al. 2001). Despite established anaerobic biodegradation potential, the natural attenuation of ethers (e.g., ETBE), and their metabolite TBA, is often slow and difficult to demonstrate in anoxic environmental conditions (Müller et al. 2007). Although natural attenuation of TBA has been demonstrated at anoxic sites, aerobic biodegradation of both TBA and ETBE is more common and rapid (e.g., Day and Gulliver 2003; Landmeyer et al. 2010).

The potential and performance of natural attenuation of organic contaminants in soil and groundwater are determined using several lines of evidence (National Research Council 1993; Alvarez and Illman 2006, 2009; Rivett and Thornton 2008; Thornton 2019):

1. Changes in the contaminant concentrations and composition (including organic metabolites and isotopic fractionation) along a plume flow path;
2. Changes in the macro-chemical composition of groundwater (e.g., dissolved oxygen, nitrate, iron, manganese, sulphate, hydrogen, carbon dioxide, and methane concentrations, collectively termed “geochemical indicators”) and general water parameters (e.g., pH, redox potential);
3. Demonstration of the presence and activity of microorganisms with contaminant biodegradation potential (often assessed using microcosm incubations or DNA and RNA analyses).

Reliable methods to detect *in situ* biodegradation of fuel oxygenates and the functioning of the microorganisms involved are crucial to underpin the first line of evidence (Nicholls et al. 2021). Isotope approaches such as compound-specific stable isotope analysis (CSIA) and position-specific isotope analysis (PSIA) have been used successfully to monitor the biodegradation of various organic contaminants, including MTBE and TBA (Rosell et al. 2007a, 2007b; Julien et al. 2015; Remaud et al. 2015; Thornton et al. 2016).

## Biodegradation of ETBE and TBA

ETBE may resist biodegradation due to its tertiary carbon atom in combination with an ether bond (Bombach et al. 2015; Thornton et al. 2020). Specific aerobic *Mycobacterium*, *Gordonia*, and *Rhodococcus* species can use ETBE as a sole source of carbon and free energy. In these strains, ETBE utilization is initiated through oxidation by cytochrome P450 monooxygenase, an enzyme encoded by the *ethB* gene within the eth*RABCD* gene cluster (Beguin et al. 2003; Schuster et al. 2013). Most microorganisms identified as aerobic ETBE degraders are members of *Actinobacteria* (Thornton et al. 2020). However, since aerobic (micro)organisms quickly deplete oxygen while mineralizing easily degradable substrates in groundwater and wastewater polluted with petroleum hydrocarbons (Grbić-Galić 1990), anaerobic ETBE biodegradation is potentially an important mechanism in groundwater. A recent review noted little evidence of anaerobic ETBE biodegradation in the presence of alternative electron acceptors, such as nitrate, ferric iron, or sulphate, or under methanogenic conditions (Thornton et al. 2020). However, anaerobic ETBE biodegradation did occur under denitrifying and methanogenic conditions, in mildly acid (~pH 5.5) soils with a low organic matter content (Yeh and Novak 1994). In anoxic microcosms with aquifer material from an industrial site, the supply of alternative ethers (ferulate, syringate, or diethyl ether) induced relatively rapid ETBE biodegradation, most likely through cometabolism (van der Waals et al. 2018). In addition, an isotope fractionation factor of up to +14‰ for hydrogen using CSIA, suggested the occurrence of anaerobic biodegradation of ETBE along a groundwater plume (Bombach et al. 2015).

Because TBA is miscible with water it has strong potential to form dissolved phase plumes in groundwater (Deeb et al. 2003; Concawe 2020). TBA is biodegraded aerobically via 2-methyl-2-hydroxy-1-propanol, 2-hydroxy isobutyric acid, 2-propanol, methacrylate, 2,3-dihydroxy-2-methyl propionate, acetone, and hydroxyacetone to carbon dioxide and water (Pedersen et al. 2022). TBA can be recalcitrant to anaerobic biodegradation (Piveteau et al. 2001; Wei and Finneran 2011). However, TBA has also been reported to biodegrade anaerobically under anoxic nitrate-, manganese-, iron-, or sulfate-reducing conditions (Mormile et al. 1994; Yeh and Novak 1994; Finneran and Lovley 2001; Somsamak et al. 2001; Bradley et al. 2002).

## Non-isotopic methods to assess anaerobic biodegradation of ETBE and TBA

### Contaminant mass loss

Contaminant mass loss in groundwater can be interpreted from related analyses using different methods. These approaches typically include (i) analysis of contaminant and geochemical indicator concentration trends in time and space, (ii) analysis of fluxes to track changes in contaminant mass, and (iii) numerical modelling of (reactive) solute transport (Alvarez and Illman 2006; Alvarez and Illman 2009; Rivett and Thornton 2008; Thornton 2019).

A decrease in contaminant concentration in groundwater over time may not result from biodegradation. It may also be due to, for example, variations in groundwater flow, dilution, or changes in contaminant source history (Gilbert 1987; Rivett and Thornton 2008). Therefore, more detailed analyses (such as mass flux estimates) or a combination of analyses indicating biodegradation is needed to deduce whether natural attenuation is occurring (Thornton et al. 2016; Thornton 2019). Mass flux analysis evaluates whether the rate at which metabolites are produced and electron acceptors are consumed equals the rate of contaminant depletion. In this analysis, the contaminant mass which passes across sections of the aquifer at different distances along a flow path is determined. The first step is to set up a quantitative model to evaluate whether mass loss occurs in the field is the creation of a conceptual site model (Alvarez and Illman 2006; Alvarez and Illman 2009; Thornton 2019). This conceptual model is based on contaminant distribution, hydrogeological conditions, and groundwater geochemistry. Available site information, such as the groundwater flow and (changes in) contaminant and geochemical indicator concentrations at various locations, should be included in this stage. Numerical models can then be developed and used to quantitatively interpret the conceptual model to determine the contaminant migration rate, estimate biodegradation rates and predict when the plume will reach a steady state (Thornton et al. 2016). However, it is often difficult to interpret differences in concentrations of ETBE and/or TBA from one monitoring well to another (Wilson and Adair 2007).

### Detection of organic metabolites

The detection of specific organic metabolites in groundwater can provide evidence of contaminant biodegradation and is useful to characterize biodegradation pathways (Thornton et al. 2016). However, quantitative evidence of *in situ* biodegradation of ETBE and TBA is difficult to obtain (Rosell et al. 2007a, b). The aerobic biodegradation of ETBE to TBA occurs via the formation of intermediate organic metabolites, including acetaldehyde and tert-butyl acetate (Thornton et al. 2020). Little is known regarding anaerobic ETBE biodegradation pathways (Thornton et al. 2020). No metabolites other than TBA have been identified (Yeh and Novak 1994; Bombach et al. 2015). However, TBA can be a primary contaminant itself, making it difficult to confirm if TBA found in an ETBE-contaminated aquifer is a metabolite from ETBE biodegradation or an original fuel component. The anaerobic ETBE biodegradation pathway(s) must be known to successfully use metabolite measurements as evidence for biodegradation. Anaerobic TBA consumption has been demonstrated, but a direct link with potential metabolites has not been described in the literature (Yeh and Novak 1994; Finneran and Lovley 2001; van der Waals et al. 2018).

### Changes in groundwater composition

Since anaerobic biodegradation of organic contaminants typically occurs at the expense of alternative terminal electron acceptors (nitrate, Fe(III), Mn(IV), sulphate, or CO_2_), it results in the accumulation of their reduced products (nitrite, Fe(II), Mn(II), sulphide, or methane). As such, changes in the geochemical composition of groundwater due to redox-driven degradation process are an important indicator of specific biodegradation processes and their extent (Alvarez and Illman 2006, 2009). Therefore, an analysis of the distribution of these geochemical indicator species along the plume flow path in time and space is important to deduce anaerobic biodegradation of ETBE and TBA, as with other organic substances in groundwater (Rivett and Thornton 2008; Thornton et al. 2016; Nicholls et al. 2021). Parameters such as redox potential and pH may also be measured as indicators of *in situ* biodegradation, although these are less important than ETBE/TBA, metabolite, and electron acceptor concentrations (Rivett and Thornton 2008; Thornton 2019). While increasing methane and sulphide concentrations along an anoxic ETBE plume could result from anaerobic ETBE biodegradation (Bombach et al. 2015), this trend may also reflect the biodegradation of co-contaminants such a BTEX compounds, potentially confounding the evidence for direct biodegradation of ETBE (Thornton et al. 2016).

### Demonstration of the presence and activity of microorganisms with ETBE or TBA biodegradation potential

DNA- and RNA-based molecular tools can be valuable in establishing the biodegradation potential of specific pollutants at contaminated sites (Lovley 2003; Babé et al. 2007). Quantitative real-time PCR (qPCR), reverse transcriptase PCR (RT-PCR), and next-generation sequencing (NGS) are now commonly deployed in the assessment of natural attenuation and enhanced bioremediation at sites polluted by organic contaminants (Alvarez and Illman 2009; Thornton et al. 2016). An increasing abundance of microorganisms with specific biodegradation capacities can, for example, be demonstrated by the quantification of 16S rRNA genes of known degrading species or genes of functional enzymes (Nicholls et al. 2021).

Cleavage of the ether bond at the tertiary carbon of ETBE may require specific microorganisms in anoxic environments (Somsamak et al. 2001; Thornton et al. 2020). Limited studies provide evidence of *in situ* anaerobic biodegradation of ETBE and TBA in soil and groundwater (Hernandez-Perez et al. 2001; Le Digabel et al. 2014; Bombach et al. 2015; Thornton et al. 2020). Possibly, microorganisms with enzymes capable of anaerobic ETBE and/or TBA biodegradation are sparsely distributed in the environment. Alternatively, the chemical and microbiological conditions necessary for biodegradation may be site-specific and sporadically developed (Thornton et al. 2020). Even if microorganisms facilitating anaerobic biodegradation of ETBE and/or TBA were present, the development of this potential may be constrained by the preferential metabolism of co-contaminants in gasoline-contaminated groundwater (Thornton et al. 2020). However, since knowledge concerning anaerobic ETBE- and/or TBA-degrading microorganisms is lacking, it is hard to provide evidence from microbiological field data to support *in situ* biotransformation. Thus, at present, it is essential to infer the potential for *in situ* biodegradation otherwise, e.g., using stable isotope methods, and combine the obtained information with other site data, such as geochemical data and microbial community compositions.

## Isotopic methods to assess anaerobic biodegradation of ETBE and TBA

### Stable isotope fractionation

Isotopes of a chemical element have the same atomic number, determined by the number of protons, but a different mass, determined by the number of neutrons. Chemical bonds containing the heavier isotope are stronger and, hence, a higher activation energy is required for their cleavage (Meckenstock et al. 2004). Therefore, chemical bonds containing the lighter isotopes are cleaved preferentially (Höhener and Aelion 2009; Hunkeler and Morasch 2009). A unique feature of isotope analysis is to obtain information on the origin and fate of compounds in the environment (e.g., Fry 2006). Assessing isotopic fractionation of contaminants in the subsurface has become a valuable tool to track biodegradation of organic pollutants in the environment (e.g., Rosell et al. 2007a, b; Julien et al. 2015; Remaud et al. 2015).

The isotopic compositions of materials are usually reported relative to an international reference standard according to the δ-notation (Table 1) (Kendall and Caldwell 1998). The Rayleigh equation (δ^13^C(*t*) = δ^13^C(0) + *ε* * ln (F)), in which δ^13^C(0) is the initial δ^13^C, δ^13^C(t) is the δ^13^C at time *t*, *ε* is the isotope enrichment factor, and *F* is the ratio of the contaminant concentrations at time *t* and time *t* (0), is used to calculate the extent of *in situ* biodegradation from measured stable isotope ratios. The isotope fractionation factor (α) indicates the change in the ratio of two isotopes during a chemical reaction or a physical process (Braeckevelt et al. 2012). Since values of α are always very close to 1, the isotope enrichment factor *ε*, defined as *ε* = (*α* − 1) × 1000, is more commonly used (Table 1) (Hunkeler and Elsner 2009). Isotope enrichment factors are obtained in controlled laboratory studies (Rosell et al. 2007a, b). By combining carbon and hydrogen isotope enrichment factors, different biodegradation mechanisms of specific contaminants can be distinguished (Zwank et al. 2005; Hunkeler and Morasch 2009).

**Table 1:** Key parameters and nomenclature used in stable isotope analysis

Parameter	Abbreviation	Equation	Used for
Isotope fractionation factor	α	$\alpha =\frac{R_P}{R_S}$ *R* = ratio heavy to light isotope *P* = product *S* = substrate (reactant)	Extent of isotope fractionation
Isotope enrichment factor	ε	*ε* = (*α* − 1) × 1000	Extent of isotope fractionation
Rayleigh equation	*δ*^13^*C or δ*^2^*H*	General form: *R* = *R*_0_ * *f* (*α* − 1) Approximate form: *δ* ≅ *δ*_0_ + *ε* * ln(*f*) *R* = isotope ratio of reactant *R*_0_ = initial isotope ratio of reactant *f* = fraction remaining *δ*_0_ = initial isotopic composition	Quantify isotope fractionation for *ε* < 20‰
Apparent kinetic isotope effect	AKIE	$AKIE=\frac{1}{1+z\ast {\varepsilon}_{rp}}$ *z* = number ‘z’ of atoms of an element at identical reactive positions *ε*_*rp*_ = isotope enrichment factor for reactive position *ε* (rp)	Differentiation specific reaction rate between the light and heavy isotope
Vienna-Pee Dee Belemnite standard for carbon	VPDB		Reference standard carbon isotopes
Vienna-Standard-Mean-Ocean-Water	VSMOW		Reference standard hydrogen isotopes

Based on Kendall and Caldwell (1998), Braeckevelt et al. (2012), and Vogt et al. (2016)

Stable isotope ratios of ^13^C/^12^C reported from the original source material or obtained from subsurface contaminant source zones are needed to differentiate isotope fractionation as a result of biodegradation. Reported δ^13^C(0) values of source contamination range from −21 to −27‰ for ETBE and from −25.5 to −31‰ for TBA, respectively (Table 2).

**Table 2:** Different isotope ratios for ETBE or TBA source material

Compound	Condition	*δ*^13^*C* (‰)	*δ*^2^*H* (‰)	Comments	Reference
ETBE	Anoxic	−25.4 to −25.8	−126 to −148	Field value	Bombach et al. (2015)
	Anoxic	−27.3 ± 0.3	−200 ± 7	Field value	Fayolle-Guichard et al. (2012)
TBA	Anoxic	−27 to −31	nd	Field value	van der Waals et al. (2018)
	Anoxic	−26.0 ± 1.0	nd	Field value	McKelvie et al. (2007)
	Anoxic	−29.0	nd	Field value	Kuder et al. (2005)
	Anoxic	−25.5 ± 0.1	nd	Field value	Zwank et al. (2005)
	Anoxic	−28.5 to −29.0	nd	Source material	Day and Gulliver (2003)
	Anoxic	−27.3 to −25.6	nd	Field value	Kolhatkar et al. (2002)

*nd* not measured/determined

The use of CSIA for the identification of *in situ* biodegradation of fuel oxygenates requires the determination of the isotope fractionation factors for specific biotic reactions (Rosell et al. 2012). The isotope fractionation during aerobic ETBE biodegradation was determined with batch experiments and different bacterial isolates to obtain stable isotope enrichment factors for carbon and hydrogen (εC, εH), (Rosell et al. 2007a, b). The isotope enrichment factors for *Aquincola tertiaricarbonis* L108 were *ε*C = −0.68 ± 0.06‰ and *ε*H = −14 ± 2‰, and for *Rhodococcus ruber* IFP 2001 were *ε*C = −0.8 ± 0.1‰ and *ε*H = −11 ± 4‰ (Table 3). The relatively low enrichment factors for both carbon and hydrogen suggest that these strains catalyse a hydrolysis reaction for ether-bond cleavage (Rosell et al. 2007a, b). Hunkeler et al. (2001) reported *ε*C = −4.2 ± 0.1‰ for aerobic TBA biodegradation. To our knowledge, there are currently no published values of *ε* for anaerobic ETBE or TBA biodegradation.

**Table 3:** CSIA studies for ETBE and TBA

Compound	Conditions	Element investigated	Lab/field	Maximum fractionation/enrichment factor	Reference
ETBE	Anoxic	Carbon Hydrogen	Field	δ^13^C = Insignificant δ^2^H = +14‰	Bombach et al. (2015)
ETBE	Anoxic	Carbon Hydrogen	Field	No degradation	Fayolle-Guichard et al. (2012)
ETBE	Oxic	Carbon Hydrogen	Lab	εC = −1.7 ± 0.2‰ εH = −73 ± 7‰	McKelvie et al. (2009)
ETBE	Oxic	Carbon Hydrogen	Lab	εC = −0.68 ± 0.06‰ −0.8 ± 0.1‰ εH = −14 ± 2‰ −11 ± 4‰	Rosell et al. (2007a, b)
TBA	Anoxic	Carbon	Field	No degradation	McKelvie et al. (2007)
TBA	Anoxic	Carbon	Field	No degradation	Kuder et al. (2005)
TBA	Anoxic	Carbon	Field	δ^13^C = +6.5‰	Day and Gulliver (2003)
TBA	Oxic	Carbon	Lab	δ^13^C = +4.3‰	Hunkeler et al. (2002)
TBA	Anoxic	Carbon	Field	No degradation	Kolhatkar et al. (2002)
TBA	Oxic	Carbon	Lab	εC = −4.2 ± 0.1‰	Hunkeler et al. (2001)

### Stable isotope probing (SIP) and bulk stable isotope analysis

One approach to investigate degradation processes is to expose microorganisms to synthesized substrates that have been enriched in a specific isotope (e.g., ^13^C). For instance, microbes could be grown in a microcosm with ^13^C-enriched ETBE or TBA. The generated microbial biomass will then have a unique isotopic signature that can be used to identify the biodegrading microorganisms, by identifying the lipids, proteins or DNA, which incorporated ^13^C during biodegradation (e.g., Dumont and Murrell 2005). This approach is referred to as Stable Isotope Probing (SIP). Further information on SIP is provided in section ‘Use of isotopically labelled compounds’.

Bulk stable isotope analysis is the determination of natural isotope ratios such as ^2^H/^1^H, ^13^C/^12^C, ^15^N/^14^N, ^18^O/^16^O, or ^34^S/^32^S of the bulk sediment and/or groundwater (Höhener and Aelion 2009). It is widely applied in ecology, but not for studies of the (bio)degradation of specific compounds (Höhener and Aelion 2009; Andersson et al. 2021). In most cases, the interpretation of bulk isotope analysis relating to specific compound biodegradation is ambiguous because it is masked by the presence of many (natural) organic compounds.

### Compound-specific stable isotope analysis

CSIA routinely uses gas or liquid chromatography coupled to isotope ratio mass spectrometry (GC-IRMS and LC-IRMS, respectively) and has become a state-of-the-art analytical method, which is being applied in many areas (Schmidt et al. 2004a, b; Thullner et al. 2012). It is used to detect the change in stable isotope ratios of a specific compound (e.g., ETBE and TBA) due to biodegradation, often resulting in the enrichment of heavy isotopes in the residual mass of those compounds (Bombach et al. 2015). CSIA can be applied to ^2^H, ^13^C, ^15^N, ^18^O, or ^34^S isotopes, depending on the substances of interest (Négrel et al. 2012). Analyses of carbon and hydrogen-isotope ratios are applied most frequently to determine organic substance degradation. Organic compounds are separated by gas chromatography during GC-IRMS and fed into a combustion oven resulting in the production of simple gases such as CO_2_ or H_2_ (Jochmann and Schmidt 2007). These are subsequently transferred to the isotope-ratio mass spectrometer (IRMS) which is targeted to the collection of specific masses. In the case of carbon, the masses of ^12^CO_2_ (44) and ^13^CO_2_ (45) are collected and quantified. Thus, possible isotope ratio shifts of carbon and hydrogen can be measured and used as an indicator for biodegradation. Aerobic and anaerobic biodegradation of MTBE was effectively discriminated by the combined use of carbon and hydrogen isotope fractionation in laboratory and field studies (Kuder et al. 2005; Zwank et al. 2005). However, no significant δ^13^C fractionation was found in an onsite pilot reactor amended with groundwater and aerobic ETBE degraders (Fayolle-Guichard et al. 2012). Similarly, no significant δ^13^C fractionation was found in MTBE at a field site with proven MTBE biodegradation potential using ^14^C assays (Thornton et al. 2011). ^13^C CSIA also produced inconclusive results on MTBE biodegradation at another polluted field site (Lesser et al. 2008). Aerobic laboratory microcosms indicated that biodegradation of 90–99% of the initial MTBE concentration resulted in a 5.5 to 6.4 ± 0.2‰ enrichment of ^13^C. The enrichment during aerobic treatment at the field site was only 25% of that found in the microcosm studies and did not deviate from the background noise in the data, although the decrease in MTBE concentration was similar (Lesser et al. 2008). Possible explanations for these observations are mixing during sampling of the groundwater, or the simultaneous occurrence of different biodegradation mechanisms *in situ* (Lesser et al. 2008; Thornton et al. 2011). Including the analysis of oxygen isotopes may improve the sensitivity of CSIA analyses for the detection of MTBE, ETBE, or TBA degradation (Kendall and Caldwell 1998). However, the determination of shifts in the ^18^O/^16^O ratios in organic molecules requires a special high-temperature combustion oven in which the organic O is specifically converted into CO, which cannot be done with current GC-IRMS equipment (Hitzfeld et al. 2017).

The interpretation of (lack of) isotope enrichment in field studies may be complicated by the presence of different initial ETBE isotope ratios in fuels from different sources (Table 2) or by the inherent microscale heterogeneity of soil and groundwater, which creates spatial variation in biodegradation potential (Thornton et al. 2011; Thullner et al. 2012). Heterogeneity may lead to local variations in isotope enrichment factors due to variations in environmental conditions and differences between the biodegrading microorganisms, as described above (Thornton et al. 2011). Hence, the variability of carbon and hydrogen enrichment factors resulting from small-scale heterogeneity and mixing during sampling must be considered when isotope patterns are interpreted, to assess biodegradation in contaminant plumes.

**Fig. 2:**
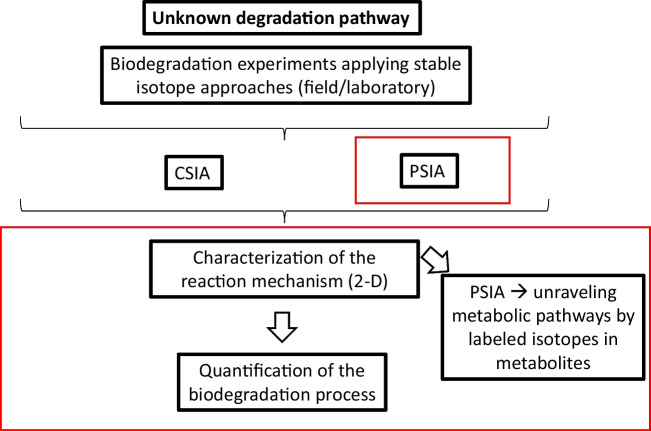
Stable isotope approaches to assess anaerobic biodegradation at ETBE- and/or TBA-contaminated sites (adapted from Vogt et al. 2016). The red rectangles indicate the knowledge gaps in the use of ETBE and TBA stable isotope approaches to determine biodegradation. Biodegradation of ETBE and TBA can be determined using CSIA and PSIA. PSIA gives additional information on the behaviour of atoms within the molecule

The application of CSIA to assess the anaerobic biodegradation at ETBE- and/or TBA-impacted sites is visualized in Fig. 2. CSIA can be used to analyse more than one element in the interpretation step (Vogt et al. 2016).

### Case studies to assess ETBE and TBA biodegradation using CSIA

CSIA has mainly been applied to assess the biodegradation of aromatic hydrocarbons, chlorinated hydrocarbons and MTBE (e.g., Meckenstock et al. 1999; Bloom et al. 2000; Thornton et al. 2011, 2016). Only two field studies examining anoxic ETBE biodegradation with CSIA are available and these showed different results (Fayolle-Guichard et al. 2012; Bombach et al. 2015) (Table 3). One study revealed no significant fractionation in carbon, while hydrogen fractionation, with respect to the most enriched ETBE found in the contaminant source zone (−126‰), was significant, with a +14‰ increase in two out of five monitoring wells with relatively low ETBE concentrations (<100μg/L), while the analytical standard deviation for hydrogen was ≤±7‰ (Bombach et al. 2015). Bombach et al. (2015) attributed the lack of significant hydrogen isotope fractionation in the three other monitoring wells to the presence of micro-environments with different ETBE biodegradation capacities that had developed along the plume flow path, similar to previous observations with MTBE (Lesser et al. 2008; Thornton et al. 2011). Another study reported no anaerobic ETBE degradation, nor significant fractionation of carbon or hydrogen along an anoxic plume, with ETBE concentrations up to 300 mg/L (Fayolle-Guichard et al. 2012). Thus, the potential use of isotope methods in ETBE and TBA biodegradation studies needs to be further investigated, especially at the field scale. As is the case for MTBE and TBA, no oxygen isotope fractionation studies for anaerobic ETBE or TBA biodegradation have been published and this is an important factor limiting the deployment of CSIA for these substances.

Several CSIA studies examining anaerobic TBA biodegradation are available (e.g., Hunkeler et al. 2001; Day and Gulliver 2003) (Table 3). The occurrence of TBA in groundwater is often associated with MTBE, which complicates studies of its fate in the field (Day and Gulliver 2003). Carbon isotope analysis can be used to establish *in situ* anaerobic or aerobic TBA biodegradation regardless of the presence of MTBE (Hunkeler et al. 2001; Schmidt et al. 2004a, b). An enrichment factor, *ε*C, of −4.2 ± 0.1‰ was determined for TBA biodegradation in aerobic cometabolic microcosms (Hunkeler et al. 2001). Anaerobic biodegradation of TBA has been inferred from the enrichment of TBA in ^13^C along a groundwater flow path (Day and Gulliver 2003). However, in other CSIA studies no evidence of *in situ* anaerobic TBA biodegradation was found (Kolhatkar et al. 2002; Kuder et al. 2005; McKelvie et al. 2007).

### Position-specific stable isotope analysis

Position-specific stable isotope analysis (PSIA), or site-specific stable isotope analysis, determines the isotopic ratio of the atoms at a particular position within a molecule. Valuable information is potentially lost in the analyses of bulk isotopic composition and CSIA since modification processes will affect the isotopic ratio specifically at the positions in the molecule where the reactions occur (Akoka and Remaud 2020). Thus, PSIA potentially provides a more sensitive measurement, by excluding non-reactive positions from the analysis (Gauchotte et al. 2009; Julien et al. 2015). Therefore, PSIA can provide more detailed and specific information to investigate the biodegradation of organic compounds in soil and water (Julien et al. 2015). Moreover, the atoms within a molecule may be enriched in ^13^C in one position, while being depleted in ^13^C at another position during a reaction (Remaud et al. 2015). Thus, the compound-specific stable isotope ratio may appear constant, while the position-specific stable isotope ratios change in opposite directions (Gauchotte et al. 2009). We are not aware of PSIA studies for ETBE or TBA focussing on ^2^H/^1^H or ^18^O/^16^O fractionation.

PSIA can be performed in various ways (Julien et al. 2015):

1. Analysis of δ^13^C by IRM-MS after chemical and/or enzymatic pre-treatment to fragment the compounds (Julien et al. 2015). If necessary, the overall compound-specific isotopic ratio can be calculated from the δ^13^C of the individual fragments.
2. Pyrolysis coupled to IRMS. Pyrolysis works well for small molecules such as MTBE. It is therefore assumed that this technique would also be effective for similar molecules, such as ETBE and TBA.
3. Quantitative ^2^H or ^13^C nuclear magnetic resonance spectrometry (NMR) can also be used for position-specific measurements. This technique has been found to be useful for the analysis of position-specific carbon isotope fractionation caused by both abiotic and biotic phenomena. Since NMR requires odd isotope numbers, it is not applicable for δ-^18^O measurements.

In a few studies, PSIA was applied to demonstrate the (bio)degradation of contaminants *in situ*. GC-IRMS combined with combustion or pyrolysis (CG-c-IRMS or GC-Py-GC-TOFMS/c-IRMS) and NMR was effectively applied to obtain position-specific carbon isotope data for various compounds, e.g., MTBE, toluene and trichloroethene (Gauchotte et al. 2009; Julien et al. 2015; Remaud et al. 2015). Intramolecular differences in the fractionation of ^13^C appear to occur during the evaporation of these compounds, as a result of variation in the interaction with water, e.g., H-bonding (Julien et al. 2015). ^13^C NMR has been used previously to determine metabolites (i.e., ethanol, TBA, 2-HIBA) formed during the biodegradation of ^13^C-labelled ETBE at micro-oxic conditions in an algal-bacterial culture (van der Waals et al. 2019). No PSIA studies on anaerobic TBA biodegradation have been found. It will be of added value to apply PSIA at field sites to detect anaerobic TBA biodegradation. Hyman (2013) noted that PSIA is a promising approach to characterize key reactions involved in the biodegradation of ether oxygenates. Besides looking at the carbon molecule, PSIA on other elements, such as hydrogen, will be interesting to perform since this can reveal additional information on biodegradation mechanisms. With NMR, the ratio of isotopes at different positions in a molecule can be determined, which makes it promising to perform PSIA for the assessment of contaminant biodegradation (Akoka and Remaud 2020).

### Limitations of isotope approaches

**Fig. 3:**
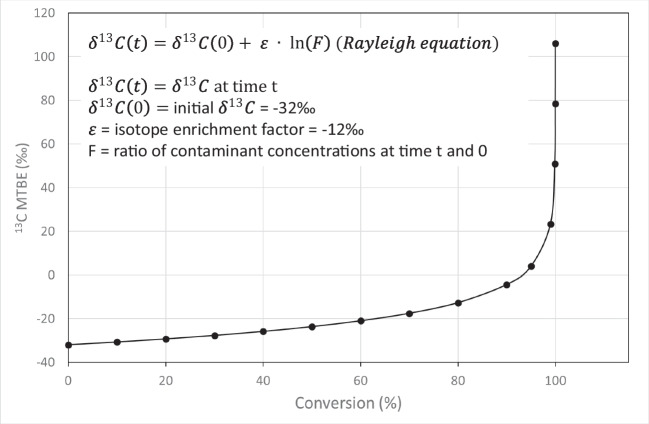
Rayleigh equation and corresponding ^13^C MTBE degradation graph

CSIA and PSIA have the potential to determine the *in situ* anaerobic biodegradation of organic contaminants, such as ETBE and TBA. In general, the isotopic fractionation process proceeds according to the Rayleigh equation, which requires significant contaminant biodegradation (e.g., >27 to 99% conversion for ETBE) for its detection using CSIA (Fig. 3) (Bombach et al. 2015). This implies that at sites where limited ETBE or TBA biodegradation has occurred additional information (e.g., hydrogeochemical conditions and the determination of predominant biodegradation pathways) is needed to confirm the results (Thullner et al. 2012; Nicholls et al. 2021).

In contrast to conventional methods to determine biodegradation (e.g., contaminant loss, change in macro-chemical parameters and presence of microorganisms with contaminant degrading potential), isotope approaches trace and detect the origin and fate of pollutants (e.g., Remaud et al. 2015). A disadvantage of isotope approaches is that both CSIA analysis using GC-IRMS and PSIA analysis using NMR have a relatively low sensitivity for hydrogen isotopes (Zwank et al. 2003; Jochmann et al. 2006; Remaud et al. 2015). If the sensitivity of CSIA could be enhanced, it could be used more often for the assessment of *in situ* biodegradation (Zwank et al. 2003). Preconcentration is a way to improve the sensitivities of CSIA and PSIA. In a thorough evaluation of sample preconcentration and injection methods, purge and trap (P&T) was found to be an efficient and reproducible concentration technique enabling the detection of 0.63 μg/L for ^13^C MTBE (Zwank et al. 2003). Next to P&T, on-column injection, split/splitless injection, and SPME were tested. The detection limit for ^2^H/^1^H ratios was 10–20 times higher than for ^13^C/^12^C, depending on the method of choice. P&T and SPME can also be used to concentrate analytes for ^18^O/^16^O analysis. Once such methods for pre-concentration are used, it should be checked whether they affect the original isotopic composition of samples (Schmidt 2003). Otherwise, non-representative results may be obtained, especially when the isotopic fractionation due to biodegradation is not very pronounced (Thornton et al. 2011). SPME, in both headspace and direct-immersion modes, was successfully applied to measure the increase of δ^13^C of MTBE and TBA during aerobic TBA biodegradation, despite small but reproducible changes of δ^13^C from the SPME and partitioning between the water and the headspace (Hunkeler et al. 2001). Current developments in using NMR pulse sequences, 2D NMR (two frequency axes), and more efficient probes can increase sensitivity, thus reducing the amount of material required for PSIA by about 10-fold (Remaud et al. 2015; Julien et al. 2016).

A second disadvantage of isotope approaches relates to the fact that these analyses do not demonstrate the activity of specific biodegradation processes at the time of sampling (Kuder and Philp 2008). Furthermore, hydrogen isotopes in alcohol molecules are often easily exchangeable with hydrogen isotopes from water. Therefore, ^2^H CSIA of alcohols, such as TBA, can yield unclear results. Hence, it is better to study other nuclei for CSIA and PSIA, such as ^13^C (Akoka and Remaud 2020). The error in the CSIA and PSIA measurements must be smaller than the variation resulting from fractionation during degradation. Fractionation is much more pronounced for H than for C, because of the relative difference of the masses which are involved (^2^H/^1^H vs ^13^C/^12^C). As a result, more accurate measurements are needed for C.

### Use of isotopically labelled compounds

Isotopically labelled compounds have been used to determine MTBE, ETBE, or TBA biodegradation and to identify the microorganisms involved. To detect MTBE or TBA biodegradation in the laboratory, the complete biodegradation to CO_2_ and/or CH_4_ can be reconstructed in microcosms that contain unlabelled growth material and are spiked with an isotopically labelled compound, e.g., ^14^C (e.g., Thornton et al. 2011). Thus, substantial MTBE and TBA degradation to ^14^CO_2_ was found under oxic, denitrifying and manganese (IV)-reducing conditions (Bradley et al. 1999; Bradley et al. 2002). Similarly, Wei and Finneran (2011) observed mineralization of TBA in anoxic incubations of fuel-contaminated sediment, with or without amendment with Fe(III) or sulphate, but reported that nitrate inhibited TBA mineralization. In contrast, no significant ^14^C-MTBE mineralization was observed in sediments regardless of electron acceptor amendment. This sensitive approach will not be useful in the field to determine TBA biodegradation since adding ^14^C-labeled substrates to the groundwater will contaminate the groundwater. Also, while the use of ^14^C-labeled substrates can indicate general potential for biodegradation of fuel oxygenates in groundwater (according to production of ^14^CO_2_), it cannot deduce the pathway involved (unless radiolabelled intermediates are sampled) or the relevant microorganisms that are responsible (Thornton et al. 2011).

Alternatively, ^13^C-labelled compounds can be used to trace biodegradation and assimilation into microbial biomass (Aslett et al. 2011; Bastida et al. 2010; Sun et al. 2012; Key et al. 2013; Bombach et al. 2015; Liu et al. 2016; van der Waals et al. 2019). During biodegradation, ^13^C-label released in intermediates (e.g., ^13^C-TBA) or end products (e.g., ^13^C-CO_2_ or ^13^C-CH_4_), indicates the catabolism of the labelled substrates. Stable isotope probing (SIP) has been successfully applied in laboratory and *in situ* microcosms and bioreactors. Through the addition of ^13^C-MTBE or ^13^C-TBA, the *in situ* activity of known aerobic MTBE- and TBA-degrading members of the order of *Burkholdariales* was confirmed (Aslett et al. 2011; Key et al. 2013). In an anoxic laboratory incubation of wastewater treatment plant samples with ^13^C-MTBE, the dominant putative degraders were classified to the family *Ruminococcaceae* and to the genus *Sphingopyxis,* belonging to the family *Sphingomonadaceae* (Sun et al. 2012). *Sphingopyxis* species are typically described as strict aerobes, but some strains appear capable of anaerobic growth with nitrate as terminal electron acceptor (García-Romero et al. 2016). SIP experiments with anoxic sediment incubated with ^13^C-MTBE confirmed the role of *Ruminococcaceae* species as MTBE-degrading bacteria (Liu et al. 2016). Such studies may provide the basis to screen ETBE- and TBA-impacted sites for microorganisms with the metabolic capability to undertake the biotransformation of these compounds (linked to the complementary functional gene assessment).

In one study, on site incubations of tubes filled with beads loaded with ^13^C-labeled TBA indicated that iron-reducing (*Geobacter* and *Geothrix*) and sulfate-reducing bacteria (*Desulfobulbus*, *Desulfovibrio* and *Desulfuromonas*) may be involved in anaerobic TBA biodegradation (Key et al. 2013). *In situ* microcosms may constitute a promising approach to assess ETBE biodegradation. *In situ* microcosms typically consist of a porous cartridge filled with sand or beads which have been loaded with a ^13^C-labelled compound such as ETBE or TBA (Bombach et al. 2010). During exposure in a groundwater monitoring well (typically 2–4 months), the microcosms are colonized by microorganisms that biodegrade the adsorbed, isotope-labelled substrate. After removal, fatty acids, DNA, or amino acids of the microbial populations that colonized the *in situ* microcosms can be extracted and identified, and their respective ^13^C/^12^C isotopic values determined. Significant ^13^C accumulation in biofilms formed in ^13^C-ETBE labelled *in situ* microcosms indicated ETBE biodegradation and assimilation into microbial biomass (Bombach et al. 2015). Fatty acid analysis indicated the activity of gram-negative ETBE-degrading microorganisms. Thus, the *in situ* microcosms allowed a sensitive detection of ETBE biodegradation at a contaminated site, but a quantitative evaluation and identification of responsible microbial species remains challenging (Bombach et al. 2015).

## Conclusion and research directions

Biodegradation, whether part of an engineered biostimulation/bioaugmentation project or a monitored natural attenuation strategy, has to be established using multiple lines-of-evidence, including (i) changes in contaminant concentrations and composition, (ii) changes in the geochemical composition of groundwater, and (iii) demonstration of the presence of microorganisms actively biodegrading the contaminants of concern. The biodegradation of petroleum hydrocarbons and methyl ether fuel oxygenates, such as MTBE and TAME, has been demonstrated using this approach as discussed above. It is however difficult to extrapolate these observations to anaerobic ETBE and TBA biodegradation due to a lack of knowledge of the microorganisms and metabolic pathways involved. While aerobic ETBE/TBA biodegradation is widely reported, it is more challenging to confidently infer anaerobic biodegradation of ETBE and TBA. The following recommendations are made to stimulate further research in key areas, deepen the current understanding of anaerobic ETBE and TBA biodegradation, and guide practitioners in the assessment and management of ETBE-impacted sites.

1. This review highlights that stable isotope techniques such as CSIA and PSIA are potentially powerful methods to detect anaerobic ETBE and TBA biodegradation. Such techniques could support assessment of quantification of *in situ* anaerobic biodegradation, in other words provide insight on the fate of ETBE and TBA in the subsurface. Nevertheless, only a few CSIA and no PSIA case studies on natural attenuation or biodegradation of ETBE and TBA are described in the literature to date. Therefore, more applications of CSIA and PSIA to ETBE/TBA plumes at field-scale are needed to determine if anaerobic biodegradation of ETBE and TBA occurs, and to estimate the respective isotope enrichment factors that enable a quantitative prediction of biodegradation to be made. If anaerobic ETBE and TBA biodegradation is occurring at a field site, it is assumed that CSIA is sufficient to demonstrate biodegradation. However, PSIA may be more sensitive than CSIA and reveal additional insight into the biodegradation mechanisms.
2. It has been shown that two-dimensional analysis of carbon and hydrogen fractionation is a powerful method to reveal pathways of MTBE biodegradation. Further insights into the biodegradation potential and mechanisms may be obtained when oxygen fractionation can be included, as highlighted in the literature. It is therefore recommended to combine CSIA and PSIA targeted at C, H, and O fractionation to evaluate ETBE and TBA biodegradation.
3. Current research suggests that anaerobic biodegradation of ETBE and TBA in soil and groundwater is not widely observed and has not been demonstrated with the limited studies undertaken. The application of CSIA and PSIA, together with molecular microbial analyses, may provide a solution to survey multiple sites to demonstrate ETBE and TBA biodegradation. A focus on sites where anaerobic biodegradation occurs would facilitate the isolation and identification of the microorganisms with ETBE and TBA biodegradation capability and the expression of their key enzymes. Thus, the ETBE and TBA biodegradation pathway(s) and key metabolites can be unravelled, and isotope enrichment factors determined. Such laboratory research forms an important basis, required to develop isotopic and molecular tools to deduce anaerobic ETBE and TBA biodegradation at impacted locations. Hence, this may enable the potential for anaerobic ETBE and TBA biodegradation to be assessed at contaminated sites via the integration of stable isotope and molecular techniques.
4. Science end-users responsible for managing ETBE/TBA impacted sites are suggested to have regard to the current limited evidence for anaerobic biodegradation of these oxygenates in developing their risk assessments and risk management strategies.
5. At the current time, it is recommended that risk managers of ETBE-impacted sites focus on monitored natural attenuation or stimulated biodegradation by maintaining or enhancing aerobic aquifer conditions (e.g., by sparging) as the most practicable *in situ* ETBE/TBA (bio)remediation approach.

## Declarations

### Disclaimer

The work reflects the views of the authors alone and may not reflect the views or opinions of Shell Global Solutions.
